# Supplementary material for: Exploring the Pathogen Profiles of Ancient Feces
Source: bioRxiv. 2025 Jan 15:2025.01.14.632942. Preprint. [Version 1] doi: 10.1101/2025.01.14.632942 (PMC11760264; doi:10.1101/2025.01.14.632942)
Supplement: Supplement 1 [file media-1.docx]

**Supplement to:**

Exploring the Pathogen Profiles of Ancient Feces

Authors: Drew Capone^1,^*^,†^, David Holcomb^2,^*, Amanda Lai^3^, Karl Reinhard^4^, Joe Brown^2^

1. Department of Environmental and Occupational Health, School of Public Health, Indiana University
2. Departments of Environmental Sciences and Engineering, Gillings School of Public Health, University of North Carolina at Chapel Hill
3. Aquaya Institute, Larkspur, California
4. Institute of Agriculture and Natural Resources, School of Natural Resources, University of Nebraska-Lincoln

**Table S1.** Primer and probe sequences for qPCR assays on the custom TAC

NOTE: Though the TAC contained RNA targets, only the DNA targets were used in this study

| **Pathogen** | **Gene** | **Primer or probe sequence (5' - 3')** | **Reference** |
| --- | --- | --- | --- |
| *Campylobacter jejuni*/*C. coli* | cadF | Fwd: CTGCTAAACCATAGAAATAAAATTTCTCAC |  |
|  |  | Rev: CTTTGAAGGTAATTTAGATATGGATAATCG | [1] |
|  |  | Probe: CATTTTGACGATTTTTGGCTTGA |  |
| *C. difficile* | tcdB | Fwd: GGTATTACCTAATGCTCCAAATAG |  |
|  |  | Rev: TTTGTGCCATCATTTTCTAAGC | [1] |
|  |  | Probe: CCTGGTGTCCATCCTGTTTC |  |
| EAEC (aaiC) | *aaiC* | Fwd: ATTGTCCTCAGGCATTTCAC |  |
|  |  | Rev: ACGACACCCCTGATAAACAA | [1] |
|  |  | Probe: TAGTGCATACTCATCATTTAAG |  |
| EAEC (aatA) | *aatA* | Fwd: CTGGCGAAAGACTGTATCAT |  |
|  |  | Rev: TTTTGCTTCATAAGCCGATAGA | [1] |
|  |  | Probe: TGGTTCTCATCTATTACAGACAGC |  |
| STEC (stx1) | *stx1* | Fwd: ACTTCTCGACTGCAAAGACGTATG |  |
|  |  | Rev: ACAAATTATCCCCTGWGCCACTATC | [1] |
|  |  | Probe: 56FAM/CTCTGCAATAGGTACTCCA/3MGB-NFQ/ |  |
| STEC (stx2) | *stx2* | F, CCACATCGGTGTCTGTTATTAACC |  |
|  |  | R, GGTCAAAACGCGCCTGATAG | [1] |
|  |  | P, 5VIC/TTGCTGTGGATATACGAGG/3MGB-NFQ/ |  |
| EPEC (eae) | *eae* | F, CATTGATCAGGATTTTTCTGGTGATA |  |
|  |  | R, CTCATGCGGAAATAGCCGTTA | [1] |
|  |  | P, 56FAM/ATACTGGCGAGACTATTTCAA/3MGB-NFQ/ |  |
| EPEC (bfpA) | *bfpA* | F, TGGTGCTTGCGCTTGCT |  |
|  |  | R, CGTTGCGCTCATTACTTCTG | [1] |
|  |  | P, 5VIC/CAGTCTGCGTCTGATTCCAA/3MGB-NFQ/ |  |
| ETEC LT | *LT* | F, TTCCCACCGGATCACCAA |  |
|  |  | R, CAACCTTGTGGTGCATGATGA | [1] |
|  |  | P, CTTGGAGAGAAGAACCCT |  |
| ETEC ST | *ST* | Fh, GCTAAACCAGYAGRGTCTTCAAAA |  |
|  |  | Fp, TGAATCACTTGACTCTTCAAAA |  |
|  |  | Rh, CCCGGTACARGCAGGATTACAACA | [1] |
|  |  | Rp, GGCAGGATTACAACAAAGTT |  |
|  |  | Ph, 6VIC/TGGTCCTGAAAGCATGAA/3MGB-NFQ/ |  |
|  |  | Pp, 6VIC/TGAACAACACATTTTACTGCT/3MGB-NFQ/ |  |
| EIEC/*Shigella* | *ipaH* | F, CCTTTTCCGCGTTCCTTGA |  |
|  |  | R, CGGAATCCGGAGGTATTGC | [1] |
|  |  | P, 56FAM/CGCCTTTCCGATACCGTCTCTGCA/3MGB-NFQ/ |  |
| *Salmonella* | *ttr* | Fwd: CTCACCAGGAGATTACAACATGG |  |
|  |  | Rev: AGCTCAGACCAAAAGTGACCATC | [1] |
|  | MGB probe | Probe: CACCGACGGCGAGACCGACTTT |  |
| *E. coli* O157 | *rfbE* | Fwd: TTTCACACTTATTGGATGGTCTCAA |  |
|  |  | Rev: CGATGAGTTTATCTGCAAGGTGAT | [1] |
|  |  | Probe: CTCTCTTTCCTCTGCGGTCCT |  |
| *Cryptosporidium* | *18S* | Fwd: GGGTTGTATTTATTAGATAAAGAACCA |  |
|  |  | Rev: AGGCCAATACCCTACCGTCT | [1] |
|  |  | Probe: TGACATATCATTCAAGTTTCTGAC |  |
| *Giardia* spp. | *18S* | Fwd: GACGGCTCAGGACAACGGTT |  |
|  |  | Rev: TTGCCAGCGGTGTCCG | [1] |
|  |  | Probe: CCCGCGGCGGTCCCTGCTAG |  |
| *E. histolytica* | *18S* | Fwd: ATTGTCGTGGCATCCTAACTCA |  |
|  |  | Rev: GCGGACGGCTCATTATAACA | [1] |
|  |  | Probe: TCATTGAATGAATTGGCCATTT |  |
| *Entamoeba* spp. | *18S rRNA* | Fwd: AAACGATGTCAACCAAGGATTG |  |
|  |  | Rev: TCCCCCTGAAGTCCATAAACTC | [1] |
|  |  | Probe: CCTTGTTCAGAACTTAAAGAGAAA |  |
| *Ascaris* | *ITS1* | Fwd: GCCACATAGTAAATTGCACACAAAT |  |
|  |  | Rev: GCCTTTCTAACAAGCCCAACAT | [1] |
|  |  | Probe: TTGGCGGACAATTGCATGCGAT |  |
| *Trichuris* | *18S rRNA* | Fwd: TTGAAACGACTTGCTCATCAACTT |  |
|  |  | Rev: CTGATTCTCCGTTAACCGTTGTC | [1] |
|  |  | Probe: CGATGGTACGCTACGTGCTTACCATGG |  |
| *Necator americanus* | ITS-2 | Fwd: CTGTTTGTCGAACGGTACTTGC |  |
|  |  | Rev: ATAACAGCGTGCACATGTTGC | [1] |
|  |  | Probe: CTGTACTACGCATTGTATAC |  |
| *Strongyloides stercoralis* | dispered repetitive sequence | Fwd: TCCAGAAAAGTCTTCACTCTCCAG |  |
|  |  | Rev: TGCGTTAGAATTTAGATATTATTGTTGCT | [1] |
|  |  | Probe: TCAGCTCCAGTTGAACAACAGCCTCCAA |  |
| *Blastocystis* spp. | 18s rRNA | Fwd: TGGTCCGRTGAACACTTTGGAT |  |
|  |  | Rev: CCTACGGAAACCTTGTTACGACTTCA | [1] |
|  |  | Probe: CTTCCTCTAAATGRTAAGATT |  |
| *Ancylostoma duodenales* | ITS-2 | Fwd: GAATGACAGCAAACTCGTTGTTG |  |
|  |  | Rev: ATACTAGCCACTGCCGAAACGT | [1] |
|  |  | Probe: ATCGTTTACCGACTTTAG |  |
| *Enterobius vermicularis* | 5S rRNA | Fwd: CAAACAACTGCATCACCAATAAC |  |
|  |  | Rev: AGTGTAGAGCAATAAGCAGTAAAG | [2] |
|  |  | Probe: TACCAACAACACTTGCACGTCTCTTCA |  |
| *H. nana* | ITS1 | Fwd: CATTGTGTACCAAATTGATGATGAGTA |  |
|  |  | Rev: CAACTGACAGCATGTTTCGATATG | [1] |
|  |  | Probe: CGTGTGCGCCTCTGGCTTACCG |  |
| enteric 16s |  | Fwd: TGCAAGTCGAACGAAGCACTTTA |  |
|  |  | Rev: GCAGGTTACCCACGCGTTAC | [1] |
|  |  | Probe: CGCCACTCAGTCACAAA |  |
| PhHV | gB | Fwd: GGGCGAATCACAGATTGAATC |  |
|  |  | Rev: GCGGTTCCAAACGTACCAA | [1] |
|  |  | Probe: TATGTGTCCGCCACCATCT |  |
| *Yersinia enterocolitica* | *lytA* | Fwd: TGATTCACCAGCAGCAATAC |  |
|  |  | Rev: GGCATCATGAAAGGCGG | [1] |
|  |  | Probe: TGTCGGTTTCTCCTTCCAGG |  |
| *Heliobacter pylori* | *ureC* | Fwd: GACACCAGAAAAAGCGGCTA |  |
|  |  | Rev: AGCGCATGTCTTCGGTTAAA | [1] |
|  |  | Probe: TCACTAAAGCGTTTTCTACC |  |
| *Plesiomonas shigelloides* | *gyrB* | Fwd: CCGCCGTGAAGGCAAAG |  |
|  |  | Rev: GCTACCGGCTCACCCAGAT | [1] |
|  |  | Probe: CACACCCAAGAATAC |  |
| *Cyclospora cayetanensi* | 18s rRNA | Fwd: AAAAGCTCGTAGTTGGATTTCTG |  |
|  |  | Rev: AACACCAACGCACGCAGC | [1] |
|  |  | Probe: AAGGCCGGATGACCACGA |  |
| *Cystoisospora belli* | 18s rRNA | Fwd: ATATTCCCTGCAGCATGTCTGTTT |  |
|  |  | Rev: CCACACGCGTATTCCAGAGA | [1] |
|  |  | Probe: CAAGTTCTGCTCACGCGCTTCTGG |  |
| *Blastocystis* spp. | 18s rRNA | Fwd: TGGTCCGRTGAACACTTTGGAT |  |
|  |  | Rev: CCTACGGAAACCTTGTTACGACTTCA | [1] |
|  |  | Probe: CTTCCTCTAAATGRTAAGATT |  |
| *Enterocytozoon bieneusi* | SSU rRNA | Fwd: TGTGTAGGCGTGAGAGTGTATCTG |  |
|  |  | Rev: CATCCAACCATCACGTACCAATC | [1] |
|  |  | Probe: CACTGCACCCACATCCCTCACCCTT |  |
| *Encephalitozoon intestinalis* | ITS | Fwd: CACCAGGTTGATTCTGCCTGAC |  |
|  |  | Rev: CTAGTTAGGCCATTACCCTAACTACCA | [1] |
|  |  | Probe: CTATCACTGAGCCGTCC |  |
| *Balantidium coli* | ITS-1 | Fwd: TGCAATGTGAATTGCAGAACC |  |
|  |  | Rev: TGGTTACGCACACTGAAACAA | [1] |
|  |  | Probe: CTGGTTTAGCCAGTGCCAGTTGC |  |
| *Acanthamoeba* spp. | 18S rRNA | Fwd: CCCAGATCGTTTACCGTGAA |  |
|  |  | Rev: TAAATATTAATGCCCCCAACTATC | [4] |
|  |  | Probe: CTGCCACCGAATACATTAGCATGG |  |

**Table S2.** TaqMan Array Card (TAC) performance and standard curve parameters**.**

| **Target** | **Target Gene** | **Slope** | **Y-intercept** | **R^2^** | **Efficiency** | **95% LOD† (copies/µL template)** |
| --- | --- | --- | --- | --- | --- | --- |
| enteric 16S | 16S | -3.309 | 38.881 | 0.998 | 101% | 1 |
| *Acanthamoeba* spp. | 18S rRNA | -3.3877 | 37.82 | 1.000 | 97% | 38 |
| *Ancylostoma duodenale* | ITS-2 | -3.3832 | 39.101 | 1.000 | 98% | 10 |
| *Ascaris lumbricoides* | ITS-1 | -3.4482 | 38.594 | 1.000 | 95% | 10 |
| *Balantidium coli* | ITS-1 | -3.3935 | 37.92 | 1.000 | 97% | 4 |
| *Blastocystis* spp. | 18S rRNA | -3.3196 | 40.64 | 0.997 | 100% | 4 |
| *Cystoisospora belli* | 18S rRNA | -3.3479 | 37.801 | 0.999 | 99% | 10 |
| *Cyclospora cayetanensi* | 18S rRNA | -3.3408 | 37.151 | 0.998 | 99% | 4 |
| *Campylobacter jejuni/coli* | *cadF* | -3.34178 | 38.27 | 0.999 | 99% | 35 |
| *Clostridium difficile* | *tcdB* | -3.4282 | 37.542 | 0.999 | 96% | 10 |
| *Cryptosporidium* spp. | 18S rRNA | -3.4033 | 37.983 | 0.999 | 97% | 1 |
| DNA control (phocine herpes virus) | *gB* | -3.315 | 37.009 | 0.998 | 100% | 10 |
| *Enterocytozoon bieneusi* | ITS | -3.2802 | 37.209 | 0.999 | 102% | 8 |
| *E. coli* O157:H7 | *rfbE* | -3.4568 | 37.976 | 1.000 | 95% | 4 |
| *Encephalitozoon intestinalis* | SSU rRNA | -3.3819 | 38.462 | 0.999 | 98% | 4 |
| *Enterobius vermicularis* | 5S | -3.4592 | 38.572 | 0.999 | 95% | 120 |
| EAEC (aaiC) | *aaiC* | -3.4241 | 38.15 | 0.999 | 96% | 10 |
| EAEC (aatA) | *aatA* | -3.4252 | 37.694 | 0.998 | 96% | 38 |
| *Entamoeba hystolytica* | 18S rRNA | -3.2775 | 37.994 | 0.996 | 102% | 10 |
| *Entamoeba* spp. | 18S rRNA | -3.2317 | 37.259 | 0.974 | 104% | 35 |
| EPEC (typical) | *bfpA* | -3.3772 | 37.465 | 0.999 | 98% | 10 |
| EPEC (atypical) | *eae* | -3.372 | 37.592 | 0.999 | 98% | 4 |
| ETEC (LT) | *LT* | -3.4638 | 47.637 | 0.990 | 94% | 485 |
| ETEC (STh) | *STh* | -3.3785 | 38.763 | 0.999 | 98% | 10 |
| ETEC (STp) | *STp* | -3.3548 | 37.266 | 0.999 | 99% | 4 |
| *Giardia* spp. | 18S rRNA | -3.4182 | 37.863 | 1.000 | 96% | 10 |
| *Hymenolepis nana* |  | -3.3804 | 38.248 | 1.000 | 98% | 4 |
| *Helicobacter pylori* | *ureC* | -3.4078 | 37.726 | 0.998 | 97% | 10 |
| *Shigella*/EIEC | *ipaH* | -3.3522 | 37.506 | 0.999 | 99% | 38 |
| *Necator americanus* | ITS-2 | -3.3686 | 39.806 | 1.000 | 98% | 7 |
| *Plesiomonas shigelloides* | *gyrB* | -3.4184 | 38.202 | 1.000 | 96% | 38 |
| *Salmonella* spp. | *invA* | -3.4191 | 38.427 | 1.000 | 96% | 4 |
| *Strongyloides stercolaris* | Dispersed repetitive sequence | -3.3316 | 37.527 | 0.999 | 100% | 4 |
| STEC (stx1) | *stx1* | -3.4083 | 39.883 | 1.000 | 97% | 120 |
| STEC (stx2) | *stx2* | -3.3697 | 38.328 | 0.967 | 98% | 160 |
| *Trichuris trichiura* | 18S rRNA | -3.3502 | 38.395 | 1.000 | 99% | 4 |
| *Yersinia enterocolitica* | *lytA* | -3.483 | 38.279 | 0.998 | 94% | 4 |

†95% LOD in gene copies per reaction calculated using methods from Stokdyk *et al*. 2016 [6]

**Figure S1. Amplification and multicomponent plots**


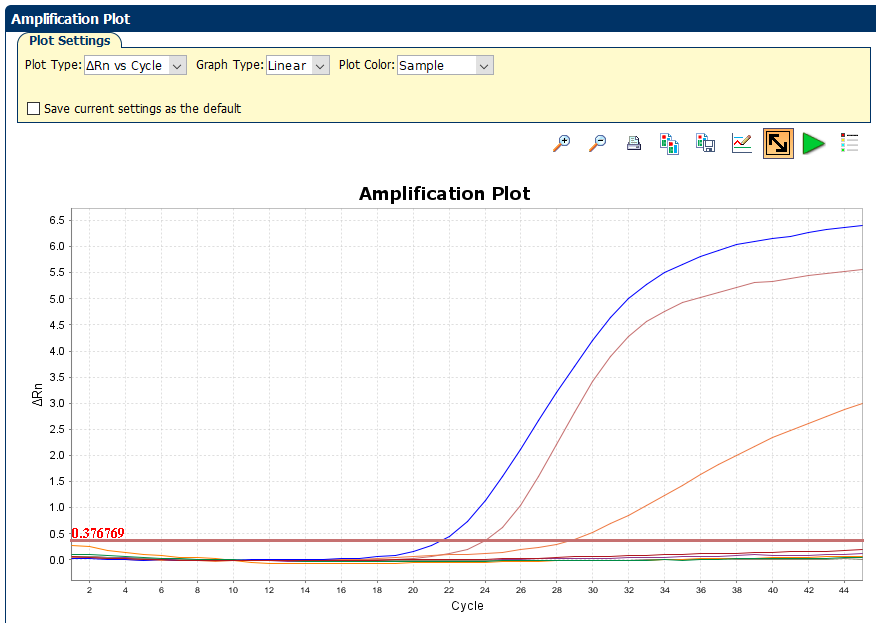


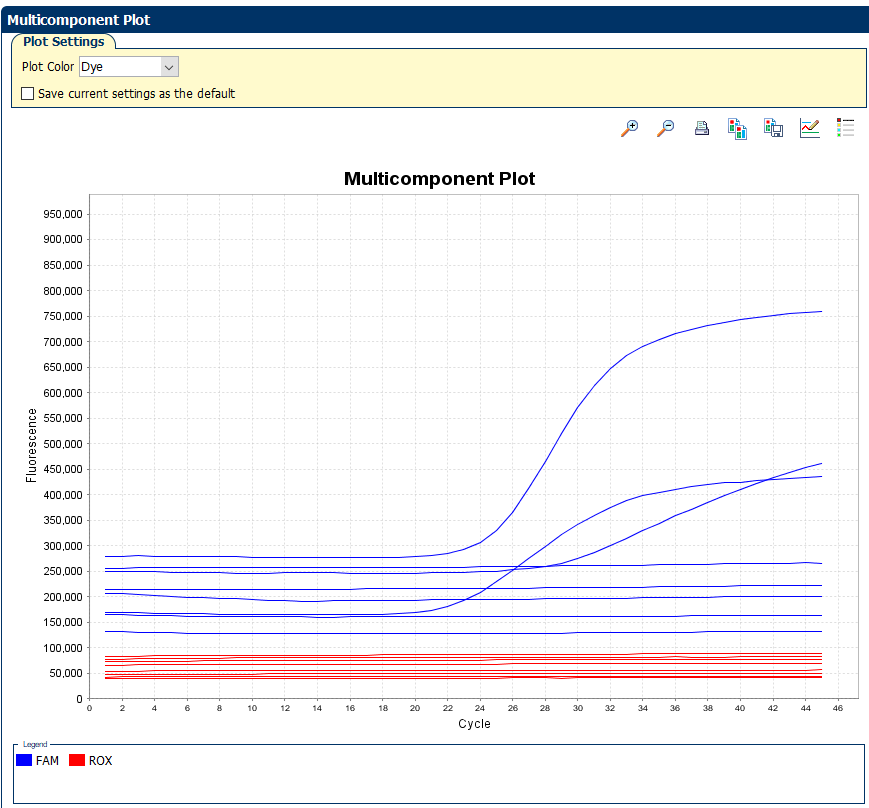


Table S3. MIQE Checklist

| **ITEM TO CHECK** | **IMPORTANCE** | **CHECKLIST** |
| --- | --- | --- |
| **EXPERIMENTAL DESIGN** |  |  |
| Definition of experimental and control groups | **E** | Cross-sectional study with no intervention or control group |
| Number within each group | **E** | 10 paleofeces from Mexico and 12 from Oregon |
| Assay carried out by core lab or investigator's lab? | D | Investigator's lab |
| **SAMPLE** |  |  |
| Description | **E** | 200 mg (see method’s section) |
| Volume/mass of sample processed | D | 200 mg |
| Microdissection or macrodissection | **E** | Not applicable |
| Processing procedure | **E** | See method’s section. |
| If frozen - how and how quickly? | **E** | Not frozen |
| If fixed - with what, how quickly? | **E** | Not fixed |
| Sample storage conditions and duration (especially for FFPE samples) | **E** | Shipped at ambient conditions. Stored at room temperature away a dark, cool, dry place |
| **NUCLEIC ACID EXTRACTION** |  |  |
| Procedure and/or instrumentation | **E** | See methods section |
| Name of kit and details of any modifications | **E** | Adapted from Hagan et al. 2020 [7] |
| Source of additional reagents used | D | Qiagen PowerBead Tubes with garnet beads |
| Details of DNase or RNAse treatment | **E** | Not applicable |
| Contamination assessment (DNA or RNA) | **E** | One extraction negative control was included during each day of extractions |
| Nucleic acid quantification | **E** | Qubit 1X HS dsDNA Kit |
| Instrument and method | **E** | Qubit 4 Fluorometer |
| RNA integrity method/instrument | **E** | Not measured |
| Inhibition testing (Cq dilutions, spike or other) | **E** | Monitored amplification of spiked controls |
| **qPCR TARGET INFORMATION** |  |  |
| If multiplex, efficiency and LOD of each assay. | **E** | Table S2 |
| *In silico* specificity screen (BLAST, etc) | **E** | We BLASTed all assays to confirm specificity before ordering the custom TAC. |
| **qPCR OLIGONUCLEOTIDES** |  |  |
| Primer sequences | **E** | Table S1 |
| Probe sequences | D** | Table S1 |
| Location and identity of any modifications | **E** | No modifications |
| Manufacturer of oligonucleotides | D | ThermoFisher Scientific |
| **qPCR PROTOCOL** |  |  |
| Complete reaction conditions | **E** | 45°C for 20 min and 95°C for 10 min, followed by 45 cycles of 95°C for 15 s and 60°C for 1 min |
| Reaction volume and amount of cDNA/DNA | **E** | 6.66 µL of template, 31.33 µL molecular grade water, 2 µL inhibition control, with 60 µL of AgPath-ID™ One-Step RT-PCR Reagents |
| Primer, (probe), Mg++ and dNTP concentrations | **E** | All assays contained the same concentrations of primers (900 nanomolar) and probe (250 nanomolar). The Mg2+ and dNTP concentrations are not listed in the in the User Guide. |
| Polymerase identity and concentration | **E** | AmpliTaq Gold™ polymerase |
| Buffer/kit identity and manufacturer | **E** | AgPath-ID™ One-Step RT-PCR Reagents |
| Additives (SYBR Green I, DMSO, etc.) | **E** | No additives |
| Manufacturer of plates/tubes and catalog number | D | ThermoFisher Scientific |
| Complete thermocycling parameters | **E** | 45°C for 20 min and 95°C for 10 min, followed by 45 cycles of 95°C for 15 s and 60°C for 1 min |
| Reaction setup (manual/robotic) | D | Manual set-up in a disinfected dead air box (10% bleach with fifteen minutes of contact time, UV for fifteen minutes, and a final cleaning step with 70% ethanol) |
| Manufacturer of qPCR instrument | **E** | ThermoFisher Scientfic |
| **qPCR VALIDATION** |  |  |
| Evidence of optimisation (from gradients) | D | See Liu *et al*. 2013 [8] and Liu *et al*. 2016 [9] |
| Specificity (gel, sequence, melt, or digest) | **E** | See Liu *et al*. 2013 [8] and Liu *et al*. 2016 [9] |
| Standard curves with slope and y-intercept | **E** | Table S2 |
| PCR efficiency calculated from slope | **E** | Table S2 |
| r2 of standard curve | **E** | Table S2 |
| Evidence for limit of detection | **E** | Table S2 |
| **DATA ANALYSIS** |  |  |
| qPCR analysis program (source, version) | **E** | QuantStudio Real-Time PCR Software V1.2 CDC |
| Cq method determination | **E** | Manual thresholding |
| Results of NTCs | **E** | Reported in the results section |
| Justification of number and choice of reference genes | **E** | N/A |
| Description of normalisation method | **E** | Normalized to mass of paleofeces |
| Software (source, version) | E | R Studio V2.2.2 |

Table S4. hCYTB484 human mtDNA primers and probe sequences.

| Oligonucleotide | Sequence (5’ to 3’) | Reference |
| --- | --- | --- |
| Fwd primer | CAATGAATCTGAGGAGGCTAC | Zhu, K.; Suttner, B.; Pickering, A.; Konstantinidis, K. T.; Brown, J. A Novel Droplet Digital PCR  Human MtDNA Assay for Fecal Source Tracking. Water Res. 2020, 183, 116085.  https://doi.org/10.1016/J.WATRES.2020.116085. |
| Rev primer | CGTGCAAGAATAGGAGGTG |  |
| Probe | ACCCTCACACGATTCTTTACCTTTCACT |  |

**References**

1. Liu J, Gratz J, Amour C, Nshama R, Walongo T, Maro A, et al. Optimization of Quantitative PCR Methods for Enteropathogen Detection. PLoS One. 2016;11: e0158199. doi:10.1371/JOURNAL.PONE.0158199

2. Rudko SP, Ruecker NJ, Ashbolt NJ, Neumann NF, Hanington PC. Enterobius vermicularis as a Novel Surrogate for the Presence of Helminth Ova in Tertiary Wastewater Treatment Plants. 2017.

3. CDC. 2019-Novel Coronavirus (2019-nCoV) Real-time rRT-PCR Panel Primers and Probes. CDC; 2020.

4. Qvarnstrom Y, Visvesvara GS, Sriram R, Da Silva AJ. Multiplex Real-Time PCR Assay for Simultaneous Detection of Acanthamoeba spp., Balamuthia mandrillaris, and Naegleria fowleri. J Clin Microbiol. 2006;44: 3589–3595. doi:10.1128/JCM.00875-06

5. Costafreda MI, Bosch A, Pintó RM. Development, Evaluation, and Standardization of a Real-Time TaqMan Reverse Transcription-PCR Assay for Quantification of Hepatitis A Virus in Clinical and Shellfish Samples. Appl Environ Microbiol. 2006;72: 3846. doi:10.1128/AEM.02660-05

6. Stokdyk JP, Firnstahl AD, Spencer SK, Burch TR, Borchardt MA. Determining the 95% limit of detection for waterborne pathogen analyses from primary concentration to qPCR. Water Res. 2016;96: 105–113. doi:10.1016/j.watres.2016.03.026

7. Hagan RW, Hofman CA, Hübner A, Reinhard K, Schnorr S, Lewis CM, et al. Comparison of extraction methods for recovering ancient microbial DNA from paleofeces. Am J Phys Anthropol. 2020;171: 275–284. doi:10.1002/ajpa.23978

8. Liu J, Gratz J, Amour C, Kibiki G, Becker S, Janaki L, et al. A laboratory-developed taqman array card for simultaneous detection of 19 enteropathogens. J Clin Microbiol. 2013;51: 472–480. doi:10.1128/JCM.02658-12

9. Liu J, Gratz J, Amour C, Nshama R, Walongo T, Maro A, et al. Optimization of Quantitative PCR Methods for Enteropathogen Detection. Chan KH, editor. PLoS One. 2016;11: e0158199. doi:10.1371/journal.pone.0158199
